# Computer Simulations of Silicide-Tetrahedrite Thermoelectric Generators

**DOI:** 10.3390/mi13111915

**Published:** 2022-11-05

**Authors:** Rodrigo Coelho, Álvaro Casi, Miguel Araiz, David Astrain, Elsa Branco Lopes, Francisco P. Brito, António P. Gonçalves

**Affiliations:** 1 C2TN, DECN, Instituto Superior Técnico, Universidade de Lisboa, Campus Tecnológico e Nuclear, 2695-066 Bobadela, Portugal; 2 Department of Engineering, Institute of Smart Cities, Public University of Navarre, Campus de Arrosadia, s/n, 31006 Pamplona, Spain; 3 MEtRICs, DEM, Universidade do Minho, 4800-058 Guimaraes, Portugal

**Keywords:** thermoelectric devices, silicide-tetrahedrite modules, computer simulations, finite element method, implicit differential method, tetrahedrites, magnesium silicides

## Abstract

With global warming and rising energy demands, it is important now than ever to transit to renewable energy systems. Thermoelectric (TE) devices can present a feasible alternative to generate clean energy from waste heat. However, to become attractive for large-scale applications, such devices must be cheap, efficient, and based on ecofriendly materials. In this study, the potential of novel silicide-tetrahedrite modules for energy generation was examined. Computer simulations based on the finite element method (FEM) and implicit finite difference method (IFDM) were performed. The developed computational models were validated against data measured on a customized system working with commercial TE devices. The models were capable of predicting the TEGs’ behavior with low deviations (≤10%). IFDM was used to study the power produced by the silicide-tetrahedrite TEGs for different ΔT between the sinks, whereas FEM was used to study the temperature distributions across the testing system in detail. To complement these results, the influence of the electrical and thermal contact resistances was evaluated. High thermal resistances were found to affect the devices ΔT up to ~15%, whereas high electrical contact resistances reduced the power output of the silicide-tetrahedrite TEGs by more than ~85%.

## 1. Introduction

Thermoelectric modules (TEMs) are devices with the ability to convert heat into usable electricity and vice versa through thermoelectric (TE) effects. Devices based on the Seebeck effect are called TE generators (TEGs), whereas devices based on the Peltier effect are called TE coolers (TECs) [1]. Usually, TEMs are solid-state devices based on sandwiched semiconductor materials (*p* and *n* types) that are electrically connected in series and thermally connected in parallel using copper or aluminum electrodes [2,3]. To thermally and electrically insulate the semiconductor legs (and the respective electrodes), the materials are normally covered by alumina or polymeric cases, which allow the devices to present multiple pairs of legs (thermocouples), designs, sizes, and heights, depending on the required applications [2]. Due to their low maintenance needs, absence of moving parts, and lack of greenhouse gas emissions, TEMs are considered ecofriendly devices [4].

In the most recent years, TEGs have become more explored and prominent, especially in the fight against global warming and climate change, as an alternative way to harvest energy from waste heat. The high potential of TEGs to generate energy from waste heat makes them interesting to apply in industries where huge amounts of low-grade heat may exist [5,6,7]. Harvesting these amounts of low-grade waste heat can allow industries, such as the cement, glass, and metallurgical industries, to not just become more efficient and sustainable, but also improve their profitability. The energy obtained by TEGs can be used to provide autonomous illumination or power small sensors and devices, reducing the energy consumed from the grid and fossil fuel consumption [8,9], and may provide energy for alarms or small communication systems. Most of the TEGs currently available on the market are based on toxic and rare elements such as Bi, Te, and Pb, which makes them expensive [10,11]. The low performance of commercial TEGs (typically < 10%) [11] compared with that of other technologies such the organic Rankine cycle [12], along with the high costs of the materials, and the low resource availability (due to the materials’ rarity) hinder the large-scale implementation of such technology. Therefore, TEGs tend to be more used in niche markets and for very specific cases or applications where the initial investment does not require an immediate or fast return [13]. With the growing need of industries and cities to switch from fossil to renewable energy sources and become more sustainable, the TEG and TEC markets are increasing every year [14]. This market growth is increasing the need to search for new and cheaper TE materials and devices, because, as noted before, most of the commercial TEMs are based on rare and toxic elements.

Among several of the materials currently being studied and explored for TE applications, such as GeTe [15], PbTe [16], half Heuslers [17], Bi_2_Te_3_ [18], and silicides [19], tetrahedrites (a copper antimony sulfosalt) have received great attention in the last decade. These materials are nontoxic, cheap, and abundant in nature, even if synthetized from their base elements [20]. They have a general formula of Cu_12-x_M_x_Sb_4_S_13_, and their compositions can be changed (e.g., x = 0.5, 1, 1.5 or 2 and with M = Ni, Mn, Cd, etc.) to improve their TE performance [20,21,22]. Tetrahedrites are type-*p* semiconductors that crystalize into a complex cubic cell (space group I-43m) that confers them the characteristic low thermal conductivity; they are bulk materials with some of the highest TE performance between 298 and 623 K [23,24,25]. Other materials with great potential for TE applications are magnesium silicides. These materials have a general formula of Mg_2_Si_1−x_ (Sn, Ge, Sb etc.)_x_, and they crystallize in antifluorite-type structures [26,27]. Like tetrahedrites, they are also constituted by low-toxicity elements that are highly abundant, and their TE performance can also be adjusted by adjusting their chemical composition.

The performance of TE materials can be evaluated through the calculation of their figure of merit, *zT*, which is a dimensionless parameter that depends on the Seebeck coefficient (S), electrical (σ) and thermal (κ) conductivities, and n the material’s absolute temperature (T). This parameter is given by $zT=\frac{S^{2}\sigma T}{\kappa }$, and materials that present values close to or higher than unity are considered to be valuable for TE applications [23]. Tetrahedrites and magnesium silicides can have *zT*’s close to unity around 623 K. Because they can operate under a common temperature range (298–623 K) [28,29,30,31], both materials are suitable for new TE devices.

Up to date and to the best of our knowledge, there are still no available silicide-tetrahedrite TEGs on the market, with the development of such devices being in its early stages. Devices based on tetrahedrites and silicides might change both industries and cities. They are highly available and inexpensive (about 1/3 of the cost of commercial Bi_2_Te_3_-based devices) if carefully designed and manufactured in high volumes, and can be more easily applied for waste-heat harvesting, even if their conversion efficiency is not high.

The first computer simulations to understand the potential of silicide and tetrahedrite materials were performed by Brito F.P. et al. [32]. The simulations, which were backed by experimental evidence, consisted of the optimization of the power output of a thermoelectric pair (or thermocouple) formed between a tetrahedrite leg (*p* type) and one magnesium silicide leg (*n* type). By using the finite element method (FEM), the authors validated the *COMSOL Multiphysics* computer-aided design (CAD) models against data from commercial devices and also experimental results obtained with a TE leg manufactured by the team. In the referred study, it was observed that a silicide-tetrahedrite thermocouple with an optimized geometry could deliver up to 0.5 W per pair. This extrapolated to a 60 × 60 mm device with and 35 pairs of elements, which produced 17.5 W and 1.6 V when the hot and cold sides of the device were exposed to 630 and 290 K, respectively.

To achieve these high voltages and high-power outputs, the authors performed an optimization procedure, and the optimal geometry involved different leg section areas for tetrahedrite and magnesium-silicide materials (7 × 7 m and 4 × 4 mm, respectively). The reason for this was that although both materials displayed comparable figures of merit, tetrahedrites were particularly suitable given their low thermal conductivity, whereas the silicides were not as good in this parameter but compensated for this deficiency by having a higher power factor. This caused the optimal section area of tetrahedrite to be larger than that of silicide, providing similar heat fluxes in both legs. At the same time, different thicknesses were evaluated for ceramic plates and copper contacts (2 and 1 mm), although the influence of these parameters was not as critical. Thus, the optimization studies mainly focused on legs sizes and spacings. Normally, commercial devices are designed with legs with equal sizes and geometries. However, in the referred work, it was observed that by independently adjusting the size of each leg, it was possible to increase the performance of the device to almost match that of commercial TEGs using expensive materials. Such optimization is related to the different properties of the TE legs. As mentioned above, because magnesium-silicide materials are electrically and thermally more conductive than tetrahedrites, by reducing their size, they can become more compatible with each other and, globally, the device can deliver more power due to an increase in the overall system thermal efficiency.

Considering the example described above, it is possible to understand that computer simulations are quite important tools for quickly and reliably predicting and studying device performance without the need to physically build and test the whole range of possible geometries. By using computational methods and models, diverse and high numbers of different cases and conditions can be analyzed, allowing the evaluation of several geometries and parameters that otherwise would require long-term and time-consuming experimentation. This example clearly shows the advantages of using computational models; these methods are becoming extensively explored by many other authors. For example, Addanki S., and Nedumaran D. [33] used computer simulations based on FEM using *COMSOL Multiphysics* software. In their study, 3D CAD models were developed to optimize the geometry of TEGs installed on handled devices. The objective was to extract the heat provided by the human touch to power small sensors or cellphones. From *COMSOL*-2D and 3D models, the authors studied the optimal parameters to manufacture multipillar TEGs with improved performance. At the same time, they validated their CAD models by testing the assembled devices under real operating conditions and by comparing the experimental data with the computer simulations. According to the findings, the multiplier TEGs delivered up to ~80 µW when a Δ*T* of 85 K was experimentally applied, confirming the reliability of the computer simulations. Similarly, Doraghi Q. et al. [34] used FEM to study the influence on the TE performance of Bi_2_Te_3_-based devices with different leg geometries. The authors compared cone- and diamond-shaped legs with the TEGs’ conventional commercial shape (rectangles). By evaluating the voltage potential through computer simulations, they verified that the diamond-shaped legs exhibited higher voltages, which was also the geometry for which the thermal stress was lower. These positive effects were caused due to the higher temperature gradients obtained across the diamond legs (probably due to an area variation), which improved the voltage potential compared with that of other shapes. In another work by Skomedal G. et al. [35], the efficiency of silicide TEGs was studied using FEM. The objective was to estimate the heat transport and total efficiency achieved by one experimental device. Their TEG prototype consisted of a set of six thermoelectric legs (silicide-based with *n-* and *p*-type elements) connected in series using molybdenum electrodes on the top and copper electrodes on the bottom. Below the Cu electrodes, a water-cooled aluminum plate was placed to work as a heat sink, while on top of the TE legs and contacts, the prototype was covered by mica and exposed to heating elements (heat source). By performing several performance tests and using the acquired experimental data, the authors evaluated the efficiency of their system and studied the effects of high electrical contact resistances on the performance of the silicide-based TEG. According to the authors, the simulations matched the experimental power when an electrical contact resistance of 2 × 10^−4^ Ω.cm^2^ was considered in their CAD model (for *n* elements exposed to the hot side). Efficiencies between 3.7% and 5.3% and powers between 1.02 and 3.24 W were achieved for temperature differences (∆*T*) ≥ 340 K.

Another powerful method of simulating thermoelectric systems is the implicit finite difference method for 1D simulations (IFDM), which can be implemented in software such as *Matlab* (from *MathWorks*, Natick, MA, USA) or *GNU Octave* (from *Octave*, San Francisco, CA, USA). This method stands out for its precision, flexibility, and low computational time, which allow it to quickly perform a huge number of simulations. Araiz et al. used the IFDM to analyze waste-heat recovery from a real industry utilizing thermoelectric generators [36]. The authors computationally optimized the occupancy ratio of one long pipe, spacing of the fins of heat exchangers, and levelized cost of energy (LCOE) of the proposed installation. Taking advantage of a 30-meter-long pipe through which gases were released at 503 K (230 °C), a maximum net power of 45 kW was achieved with an occupancy ratio of 0.40 and a fin spacing of 10 mm. Martinez et al. studied a thermoelectric self-cooling device by utilizing the IFDM to simulate the transient and steady state of the whole system utilizing *Matlab* [37]. The data obtained with the computational system were compared with those obtained from an experimental device. Statistical studies indicated a maximum deviation of 12% between the experimental and simulated values for the main outputs. In addition, the model was able to predict the behavior of the systems under abruptly changing conditions. The findings displayed the ability of the finite difference method to simulate the performance of TE devices with small deviations.

In the present work, the potential of novel TE devices based on tetrahedrites and magnesium silicide thermoelectric materials was evaluated using two different computational methods. One was based on the 3D finite element method (FEM) by utilizing *COMSOL* software, which can provide huge amounts of information about the performance of TE devices working under particular conditions. The other was based on the implicit 1D finite difference method (IFDM) and was coded in *Matlab*, which is a more suitable method for simulating several conditions, as the computational time required for a singular case is extremely low (<30 s). The two computational methods and their respective computer models were validated through the experimental data obtained from a custom-built set up working with commercial TE devices. Then, the two methods were used to simulate the novel silicide-tetrahedrite TEGs. The 1D method was utilized to simulate many working conditions of the TE device to obtain a bigger picture of the performance of the novel TE materials. The 3D FEM was used to perform a refined analysis of the performance of the newly developed materials under experimental working conditions. This work allowed identifying the main advantages and key features of the development of a new generation of efficient and affordable devices based on silicide-tetrahedrite TEMs.

## 2. Experimental Setup, Computational Methods, and Models

### 2.1. TEG Testing System

**Figure 1:**
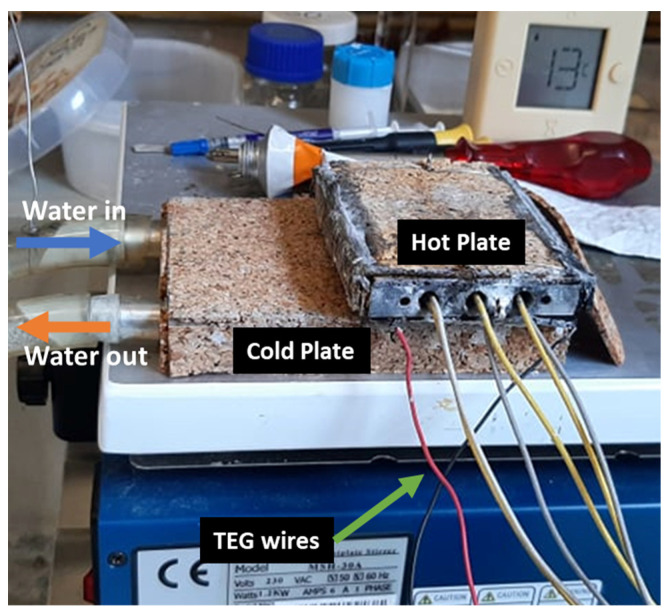
Experimental set up.

The TEG testing system is presented in Figure 1 and is based on three main elements: one aluminum heating block, provided with three electrical cartridges (175 W each; model H6338S175A, from *Ivaldi*, Eveux, Normandy, France); the TEM (GM200-49-45-30, from *European Thermodynamics*, Leicestershire, United kingdom); and a heat sink consisting of a water-cooled heat exchanger for the cold side, which used tap water to dissipate the heat that passed through the TEM.

The heating cartridges inserted in the aluminum heating block were connected in parallel to an electrical DC power supply (*VARIAC^®^* 270V-8A, from Cleveland, USA that was used as the heat source of the set up. The temperature of the hot face of the module was changed in each test by increasing the heat flux through the adjustable power supply. The TEM was located between the heating and cooling blocks to force a thermal gradient between the hot and cold faces. Thermal paste (HY410, 1.42 W/m.K, *Halnziye Electronics*, Shenzhen, China) was used on both faces of the TEM to ensure proper contact and minimize the thermal contact resistance between the module and heat exchangers. Lastly, pressure was manually applied to the TEM to ensure that good contact was provided at both faces. To keep all the set-up blocks in contact and reduce the thermal resistance, two additional weights with a total weight of ~3 kg were placed on the top of the system.

The water-cooled heat exchanger used tap water to cool down the TEM, and the water flow was maintained constant during all the tests to ensure similar working conditions and a similar thermal resistance of the cold side. In addition, all the aluminum blocks were insulated with cork (2 mm thickness) in order to minimize the heat lost to the ambient environment.

The TEG measuring equipment was composed by a type-K thermocouple rod inserted in the aluminum heating block to measure the temperature of the heat exchanger, and another type K thermocouple was introduced in the outlet of the water-cooling stream to obtain the temperature of the heat sink. Both thermocouples were connected to a dual-probe digital thermometer (model *Fluke 52 k/j* thermometer, from *Fluke^®^*, Everett, WA, USA) to measure the separate temperatures and gradients. In addition, an *I-V* tracer (*RO3-series* from *TCS*, Glasgow, UK) was used to collect ad evaluate the power output produced by the TEM. The *RO3* rapidly and automatically varied the load resistance to obtain the maximum possible power output of the system and extract data. The data collected by the *RO3* device were then imported to a personal computer (*Hewlett-Packard*, Palo Alto, CA, USA) and managed in a *Microsoft excel* spreadsheet (Software from *Microsoft*, Redmond, WA, USA).

The TEG tests started with gradual increases in the voltage of the power supply of the heat source (10 by 10 V or more) with a constant water flux; after temperature stabilization (+/−30 min), the load resistance was automatically and rapidly varied by using the *RO3* device for data acquisition. Then, the temperatures of the heat source and sinks were registered, and the process was repeated until the temperature of the heat source matched the TEM maximum allowable working temperature (~473 K).

### 2.2. Finite Element Method (3D)

**Figure 2:**
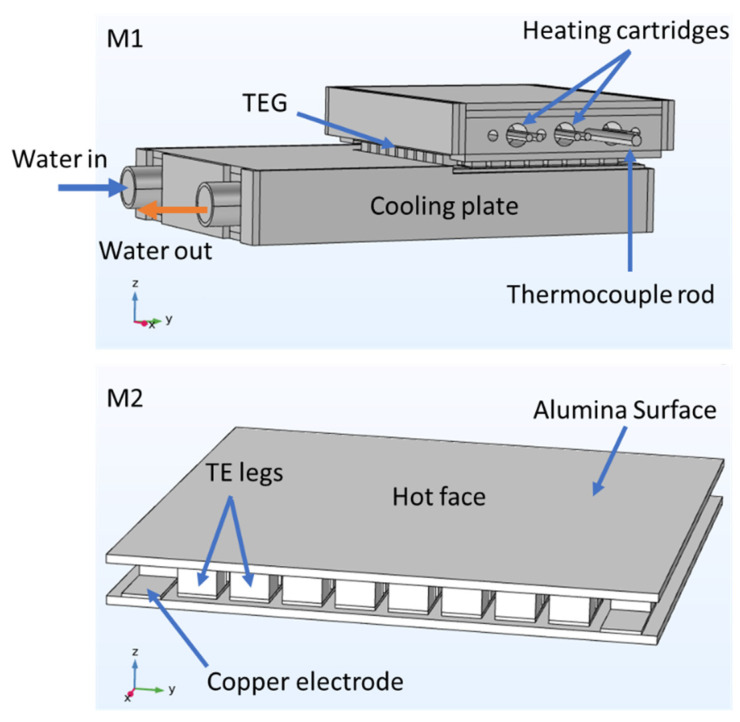
The 3D CAD geometries used to simulate the whole testing system (M1) and a TE module (M2).

The simulations using FEM were performed with *COMSOL Multiphysics* software (from *COMSOL*, Stockholm, Sweden). The method is based on the use of 3D CAD models, where several mesh elements can be built and applied. To these meshes, usually described as sets of discretized elements and nodes, several differential equations are defined along with specific boundary conditions that are used to simulate parts, devices, and/or processes. For modeling the experimental testing system using the FEM, two separate 3D CAD models were developed. The first CAD model (called M1) was dedicated to the global system, where the temperature gradients could be evaluated and extracted. The second CAD model (called M2) was dedicated to evaluate the TEG performance, where the typical current voltage (IV) and current power (IP) curves could be obtained using the temperatures simulated in M1. The CAD geometry for both 3D models (M1 and M2) is shown in Figure 2.

The equations used for the modeling of the heat transfer in the TEMs and the testing system are based on Fourier’s law, defined as:

(1) $$\rho c_{p}\cdot \nabla T+\nabla \cdot q=Q+Q_{ted}$$

(2) q=−κ∇T

where ρ is the density, $c_{p}$ is the heat capacity at constant pressure, T is the temperature field, q is the heat flux by conduction, κ is the thermal conductivity, $Q_{ted}$ is the thermoelastic damping, and Q is an additional heat source. Equations (1) and (2) were applied to M1 and to M2.

The three heating cartridges that allowed the temperature control of the heat source (inserted on the top aluminum plate) were defined in the simulations as the heat sources of the system (Figure 2, model M1). The heat rate was calculated through:

(3) $$Q_{0}=\frac{P_{0}}{V}$$

where $Q_{0}$ is the heat source, $P_{0}$ is the heat rate, and V is the volume. Because the experimental testing system was insulated with cork, the system was considered thermally insulated in all simulations, except for the aluminum -exposed surfaces. On these surfaces, heat losses by radiation to the ambient air were considered, defined as:

(4) $$-n\cdot q=\varepsilon \sigma \left(T_{Amb}^{4}-T^{4}\right)$$

where ε is the aluminium surface emissivity, σ is the Stefan–Boltzmann constant, n is the surface normal, and $T_{Amb}$ is the ambient temperature.

For the simulation of the water flow through the heat sink pipes, the laminar regime was assumed. In *COMSOL*, the modeling of the laminar flow regime is based on the Navier–Stokes equations, where the motion of fluids for specific boundary conditions such as the inlet, outlet, and model walls can be defined as:

(5) ρ(u·∇)u=∇·[−pI+K]+F+ρg 

(6) ρ∇·u=0

(7) $$K=\mu (\nabla u+{\left(\nabla u\right)}^{T})$$

where ρ is the fluid density, p is the fluid pressure, μ is the fluid viscosity, F is the volume force, I is an identity matrix, g is the gravity force, and K is the viscous forces in the fluid. In summary, Equation (5) is a momentum balance from Newton’s second law, whereas Equation (6) can be considered an equation of continuity, which represents the conservation of mass considering fluid incompressibility [38]. Equations (6) and (7) were only applied to M1 because fluids were not considered in M2.

For the simulation of the TEG inserted between the heat exchangers, the following equations were applied:

(8) $$\nabla \cdot J=Q_{j,v}\;$$

(9) $$J=\sigma E+J_{e}\;$$

(10) E=−∇V

The presented equations are based on Ohm’s law, where σ represents the electrical conductivity, $J_{e}$ is the current density (externally generated), J is the current density, E is the electric field intensity, and is $Q_{j,v}$ the current source, also called the volumetric current source.

To account for the electrical contact resistance between the TEG legs and copper electrodes, the interfacial resistance was modulated using the following equations:

(11) $$n\cdot J_{1}=\frac{1}{\rho_{1}}\left(V_{1}-V_{2}\right)\;$$

(12) $$n\cdot J_{2}=\frac{1}{\rho_{2}}\left(V_{1}-V_{2}\right)\;$$

where ρ is the legs’ surface resistance, V is the voltage, J is the current density, and n is the surface normal. Numbers 1 and 2 refer to the two sides of the boundary (top and bottom of the legs’ contact interface, respectively). In summary, Equations (11) and (12) relate the electric current density to the jumps in the electric potential, allowing the manual definition of specific contact resistances or to study the contact resistance of devices trough iteration processes based on experimental data (IV and IP curves).

To modulate the effects of the TE and Joule effect (present at the TE legs and on the copper electrodes), the following equations were applied:

(13) P=ST 

(14) $$\mu_{Th}=T\frac{dS}{dT}\;$$

(15) q=−κ∇T+PJ

(16) J=−σ(∇V+S∇T)

where P represents the Peltier coefficient, S is the Seebeck coefficient, $\mu_{Th}$ is the Thomson coefficient, q is the conductive heat flux, J is the electric current density, κ is the thermal conductivity, and σ is the electrical conductivity. To use Equations (13)–(16) and properly simulate the electrical circuit of the TE modules, specific electronic components needed to be considered or emulated. They were as follows: one load resistance, one ground component, and one circuit terminal for the voltage readings.

In summary, the simulations were performed in a stationary state, with parametric sweep studies performed to solve the CAD models. For the parametric sweep for M1, the input parameters used for solving the model were as follows:

- Water mass flow rate (kg/s);
- TEG surface thermal resistance (top and bottom) (K.m^2^/W);
- Electrical contact resistance on the TE elements junctions (Ω.m^2^);
- Ambient temperature (K);
- Water inlet Temperature (K);
- Heat source power, or power range used (W);
- Load resistance (Ω).

The output of M1 was the 3D temperature profile of the testing system. For M2, the main input parameters used in the parametric study were:

- TEG hot-side temperature (retrieved from M1) (K);
- TEG cold-side temperature (retrieved from M1) (K);
- Load resistance range (equal to M1) (Ω).

The outputs of M2 were the IV and IP curves of the simulated devices (inserted between the heat source and heat sinks).

All the materials properties necessary to perform the simulations were taken from the *COMSOL* materials database and from other works [32,39,40]. The meshes (built on M1 and M2) were customized, being finer in the M1 model at the tube walls, at the TEG copper electrodes zone, and at the heating cartridges tubes. In M2, the meshes were more refined only at the TEG copper electrode zone. The customization of the meshes allowed us to reach a balance between accuracy and computation time while ensuring practical and reliable simulations. The validation of 3D CAD models presented in the next sections was performed through an interactive process using the experimental data as the model inputs.

### 2.3. Implicit Finite Difference Method (1D)

The IFDM was used to simulate the behavior of the TEG that harvested waste heat from a heat source, obtaining the power generated by the TEMs, heat flow, and temperature distribution across the entire system. The model solved the thermal–electrical analogy of the TEGs, with each element being introduced as a thermal resistance.

**Figure 3:**
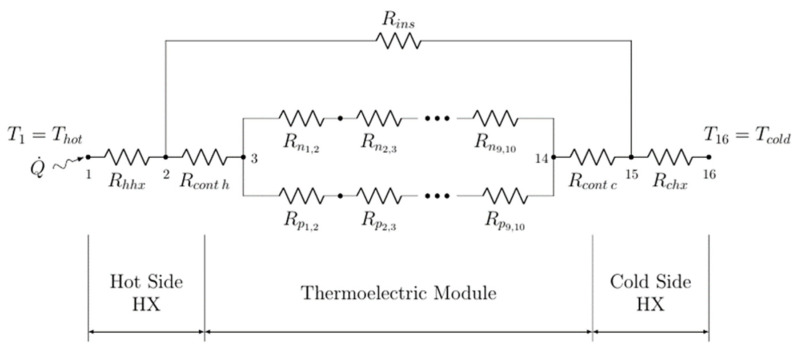
Schematic of the thermal resistances of the simulated TEGs.

The model is based on a previously published model that was adapted for this application with a fixed heat source and heat sink temperature. It was coded in *Matlab* under the assumption of unidirectional heat transfer. The system was discretized in 16 nodes, as shown in Figure 3. It included the heat source, heat sink (ambient), heat exchangers (hot and cold sides), and TEMs (junctions, ceramics, and thermoelectric materials).

The heat source was represented by node 1 and the ambient air by node 16. The hot-side heat exchanger corresponded to $R_{hhx}$, while the cold-side heat exchanger was represented by $R_{chx}$. $R_{cont\;h}$ and $R_{cont\;c}$ are the thermal contact resistances of the cold and hot sides of the TEM, respectively, which were introduced as a fixed value taken from the experimental set-up (0.00016 K.m^2^/W, thermal grease HY410, Halnziye Electronics, Shenzhen, China).

Nodes 3 to 14 represented the TEM and all the thermoelectric phenomena (Peltier, Seebeck, Thomson, and Joule effects), which, along with the Fourier law, were considered as follows:

(17) $$\propto_{AB}=\frac{dE}{dT}=\propto_{A}-\propto_{B}\;$$

(18) $${\dot{Q}}_{Peltier}=\pm \pi_{AB}I=\pm IT\left(\propto_{A}-\propto_{B}\right)\;$$

(19) $${\dot{Q}}_{Thomson}=-\sigma \vec{I}\left(\vec{\Delta T}\right)$$

(20) $${\dot{Q}}_{Joule}=R_{0}\left(I^{2}\right)$$

(21) $$\rho c_{p}\frac{\partial T}{\partial t}=k\left(\frac{\partial^{2}T}{\partial x^{3}}+\frac{\partial^{2}T}{\partial y^{3}}+\frac{\partial^{2}T}{\partial z^{3}}\right)$$

The TEM was represented by 11 thermal resistances: 2 corresponded to the ceramic plates of the cold and hot sides, 2 represented the union between the thermocouples on the cold and hot sides, and each thermoelectric leg was discretized in 6 thermal resistances by dividing the leg into 7 equal length segments. The model also took into account the thermal bridge that appeared between the heat source and heat sinks, represented as $R_{Ins}$. It also predicted the optimum electric load resistance that maximized the power generated with the TEM, similar to a maximum power point tracking (MPPT) device, by varying the load resistance and then selecting the maximum power output, if needed. The main inputs needed to solve the model were:

- Heat source temperature (K);
- Heat sink temperature (K);
- Range of load resistance for the TEM values (Ω);
- Number of TEMs in the generator/system (#);
- Thermal resistance of the cold-side heat exchanger (K/W);
- Thermal resistance of the hot-side heat exchanger (K/W);
- Electrical resistance of the cables used (Ω);
- Dimensions of the TEM (ceramic, unions, legs, etc.);
- Thermal conductivity of the TEM materials (W/m.K) as a function of the temperature (f(T));
- Electrical resistivity of the TEM materials (Ω.m) as a function of the temperature (f(T));
- Seebeck coefficient of the TEM materials (V/K) as a function of the temperature (f(T));
- Thermal conductivity of the union material (W/m.K);
- Electrical resistivity of the union material (Ω.m);
- Thermal conductivity of the ceramic material (W/m.K).

With the aforementioned inputs and through an iterative process, the model was able to calculate the behavior of the system. The main outputs obtained with the model were the following:

- Temperature at each node (K);
- Heat flux across the system (W);
- Power generated by the TEMs (W);
- Efficiency of the TEMs (%).

## 3. Results and Discussion

### 3.1. Model Validation

**Figure 4:**
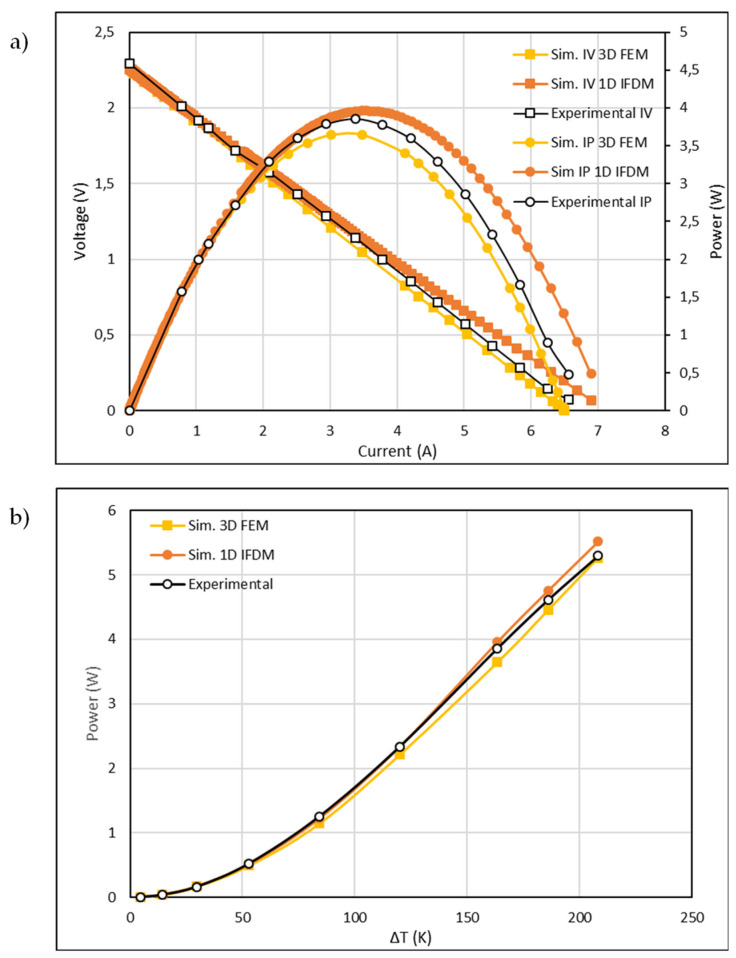
(**a**) Typical IV and IP curves for the maximum power obtained when the sink Δ*T* was 163 K, and (**b**) power vs. temperature difference (heat source and sinks) for the GM200 simulated and tested in the experimental set up.

First, in order to probe the reliability of the methods, a validation of the computational models was performed. For that, the experimental and computational results were compared, and the power generated as a function of the thermal gradient and the IV curves were generated, as shown in Figure 4. In Figure 4a, the typical IV and IP curves for the maximum power output of the TEG while operating at a sink ΔT of 163 K is presented. Figure 4b compares the power vs. temperature difference obtained for the GM200 commercial module and shows that the computer simulations (performed by the two methods) fairly predicted the power generated by the GM200 device at all the tested temperatures.

As shown in Figure 4b, at the highest Δ*T*, the power output reached ~5.3 W, which corresponded to a maximum voltage of around 2.7 V and showed strong agreement between the experimental and simulated data. In summary, both models were capable of predicting the experimental voltages and powers, with slight deviations always between the ±10% data interval. Taking this into consideration, we concluded that the displayed data proved the reliability of the developed computational methods and models, showing that they can be used to predict the power of TE devices while operating under similar laboratory conditions.

The computational model based on FEM 3D and developed in *COMSOL* produced a reliable tool that is suitable for predicting the behavior of a TEG and providing precise information on the temperature and heat distribution in 3D and 2D environments. The main disadvantage of this model is the long computational time required to solve the system, which limits the potential of the model when studying large numbers of working conditions, dimensions, load resistances, etc. Although the 1D implicit finite difference method developed in *Matlab* does not provide 3D information on the system, it stands out due to the low computational time needed to obtain the performance of the TEM under the designated working conditions. This feature of the 1D IFDM model complements the main disadvantage of the 3D FEM model. Therefore, the combination of both models to simulate a TE device provides plenty of information to assess the strengths, weaknesses, and viability of new TE devices and their operational environments.

### 3.2. Simulations of the Novel Silicide-Tetrahedrite Devices

First, simulations of the new silicide-tetrahedrite devices were performed using the IFDM 1D model to determine the potential of these new materials. Then, FEM 3D models were used to analyze the increase in the thermal efficiency of different configurations of the thermoelements and the impact of the thermal and electrical contact resistances in harvesting power.

#### 3.2.1. IFDM 1D Results

The IFDM 1D model was used to predict the potential of a silicide-tetrahedrite TEM working as a TEG. Two different configurations of the silicide-tetrahedrite device were tested in terms of disposition, dimensions, and number of legs of the thermoelectric materials. Both cases were analyzed for a 60 × 60 mm module with an alumina thickness of 0.9 mm. The first case corresponded to the same configuration of thermoelements as the GM200 module, which consisted of 49 thermocouples with equal dimensions of the *p* and *n* legs, with a section of 4x4 mm and a height of 3 mm. The second case corresponded to a thermally optimized distribution in which both legs did not match in size, and with 35 thermocouples. In this last case, the section of the *p* element was 7 × 7 mm whereas the *n* element remained at 4 × 4 mm, and the height remained at 3 mm. Both cases are simulated alongside the GM200 device as a comparison point.

The temperature of the cold face was set to 20 °C for all cases, while the hot face temperature varied. For the GM200 device, the temperatures of the hot face were simulated from 50 to 200 °C (maximum working temperature) in 10 °C increments. For the silicide-tetrahedrite device, in both cases (i) and (ii), the hot face temperature started at 50 and increased up to 300 °C (maximum operating temperature of the materials) in 10 °C increments. The load resistance was varied for each simulation until the maximum power output was obtained for each temperature gradient between the faces.

**Figure 5:**
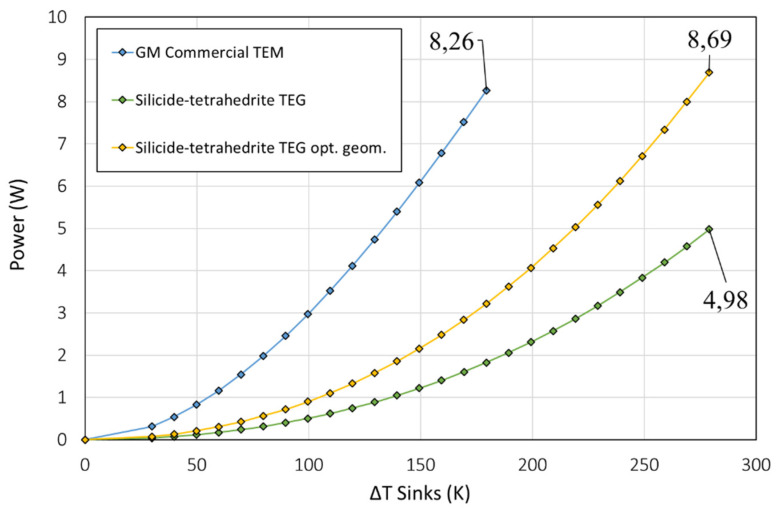
Power as a function of the Δ*T* between sinks (IFDM 1D).

In Figure 5, the power generated with the TEM is represented as a function of the temperature difference between the heat sink and source. The blue line (top) corresponds to the commercial GM200 thermoelectric module, the green line (bottom) corresponds to the configuration of the commercial GM200 module with silicide-tetrahedrite material properties, and the yellow line (middle) corresponds to the optimized distribution with silicide-tetrahedrite material properties.

The commercial GM200 module was able to achieve a power output of 8.26 W at a hot-side temperature near 200 °C (maximum operation conditions). When the properties of the GM200 were replaced with silicide-tetrahedrite, and the dimensions of the module were maintained, the maximum power output drastically dropped to 4.98 W at a hot-face temperature of 300 °C (maximum operation conditions). However, when the dimensions of the TEM using silicide-tetrahedrite properties were replaced with a previously analyzed optimized design [32], the power output of the new materials was able to surpass that of the commercial module with 8.69 W at a hot-side temperature of 300 °C. It is important to highlight that the higher temperature gradient of the silicide-tetrahedrite properties is possible due to the capabilities of these materials to withstand such temperatures in comparison with bismuth-telluride.

**Figure 6:**
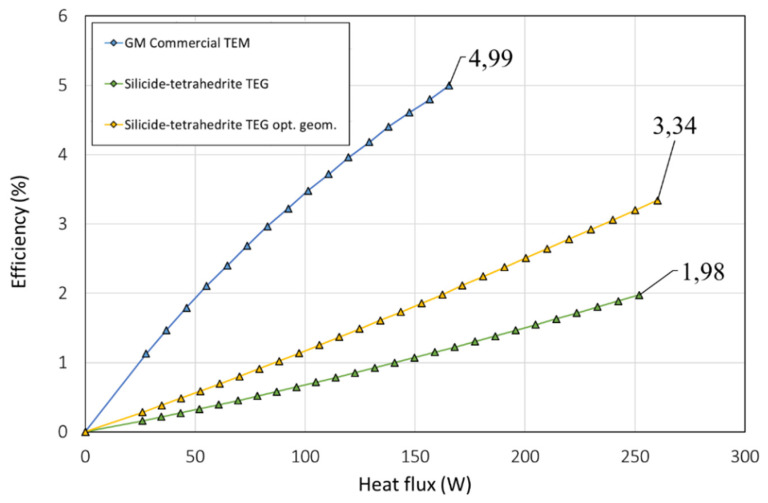
Efficiency of the TEM as a function of the heat flux (IFDM 1D).

Figure 6 represents the efficiency of the TEMs as a function of the heat flux that crossed the devices. Is clear that the GM200 bismuth-telluride device outperformed the silicide-tetrahedrite materials in terms of efficiency due to the superior *zT* of the materials. Regardless, the figure also depicts the relevance of the optimization of the dimensions of the TEM in the final performance of the device for the same thermoelectric properties. For the same hot-side temperature, the maximum efficiency obtained with the not-optimized design was 1.98%, whereas with the optimized dimensions, the performance of the TEM was boosted by 68%, achieving an efficiency of 3.34%.

In summary, the bismuth- telluride commercial GM200 module still outperformed the silicide-tetrahedrite devices due to the greater *zT*. However, despite the larger *zT* of the bismuth telluride module, the increased range of working conditions of the tetrahedrites and magnesium silicide materials for higher temperatures significantly improved their viability, so they can achieve greater maximum power outputs than the commercial bismuth-telluride modules and at a fraction of the cost. Lastly, we note the advantages of the developed tetrahedrites and silicides devices in terms of environmental benefits, viability, and materials availability compared with the bismuth and telluride ones.

#### 3.2.2. FEM 3D Results

The FEM 3D method was used to complement the simulations produced by the IFDM 1D method displayed above. These simulations consisted of analyzing the temperature distribution on the testing system when working using three simulated devices: the Bi_2_Te_3_-based TEM (GM200), silicide-tetrahedrite device with optimized geometry, and silicide-tetrahedrite device with conventional geometry. To simulate the temperature profile of the testing system when working with the silicide-tetrahedrite device with the optimized geometry, two additional 3D CAD models were designed. The new models, called M3 and M4, can be observed in Supplementary Materials Figure S1, with the geometry specifications for M4 presented in Table S1.

**Figure 7:**
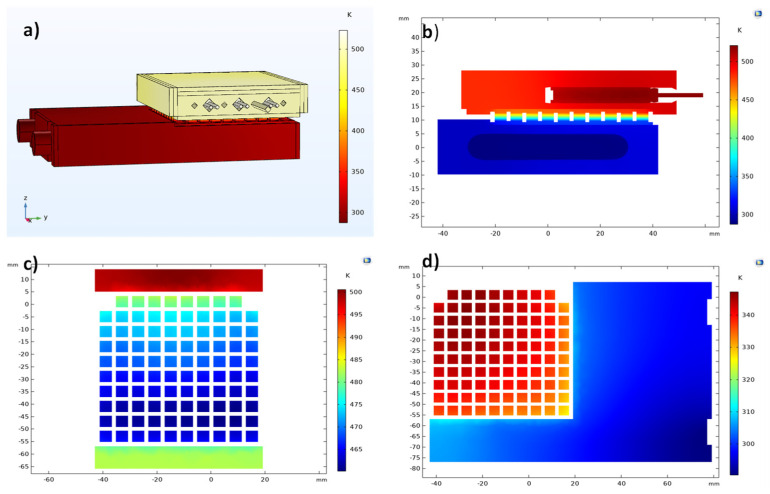
(**a**) Temperature distribution across the testing system using the GM200 device; (**b**) temperature distribution from a vertical plane cut taken at the middle of the plate; (**c**) temperature distribution on the top of the TEG legs (hot zone); (**d**) temperature distribution on the bottom of the TEG legs (cold zone).

The obtained temperature profile of the GM200 TEG working on the testing system is presented in Figure 7. The simulation input parameters were based on the experimental testing of the real device, where we used a heating cartridge power of 204.6 W, a water flux of 0.0101 kg/s, a thermal resistance of ~0.0038 K.m^2^/W (at both sides), and an electrical contact resistance of 15 mΩ.mm^2^. In Figure 7a, the global system temperature can be observed; Figure 7b displays a 2D cut taken at the middle of the testing system. Lastly, the temperature profile of the TEG legs (top and bottom) is shown on the 2D horizontal cuts in Figure 7c,d.

As shown in Figure 7, the temperature distribution at the TEG legs was not homogeneous. The observed temperature differences were caused by the testing system geometry or, more specifically, by the configuration of the heating and cooling plates that accommodated the heating cartridges and the water cooling pipes. As the heating plate shifted to the right (Figure 7a), the heat was more easily removed by the heat sink for the TE legs positioned on the left side. This geometry configuration made the TE legs of the module to present different Δ*Ts* depending on their disposition (left or right). Despite how this temperature difference affected the performance of the tested devices being unclear, there is a chance the power output may be reduced by temperature inhomogeneities.

**Figure 8:**
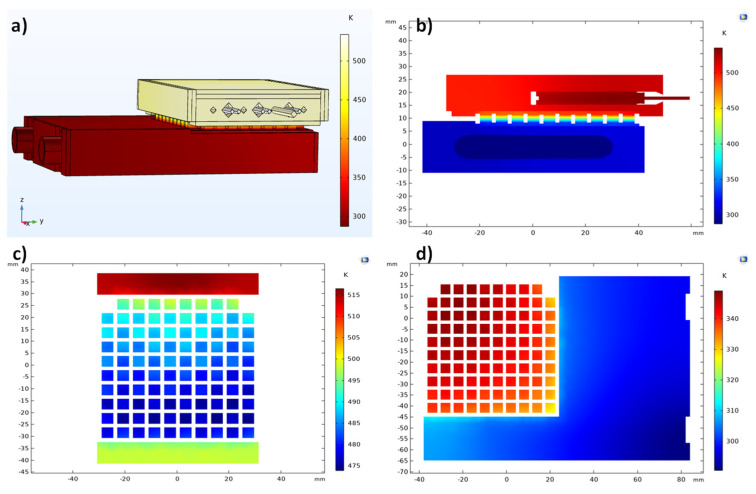
(**a**) Temperature distribution across the testing system when using the silicide-tetrahedrite TEG with a conventional geometry; (**b**) temperature distribution from a vertical plane cut taken from the middle of the plate; (**c**) temperature distribution on the top of the TEG legs (hot zone); (**d**) temperature distribution on the bottom of the TEG legs (cold zone).

The temperature profile simulation of the silicide-tetrahedrite device with conventional geometry is displayed in Figure 8. As can be observed, the temperature distribution is similar to that presented in Figure 7. Although, when observing Figure 8c in detail, it is possible to notice that some TE legs were slightly hotter in regions where their temperature should have been almost similar. This effect could be explained by the different thermal conductivity between tetrahedrite and magnesium silicide materials (~0.6 and ~2.7 W/m.K around 450 K, respectively) [32]. Because the silicide legs were more thermally conductive, they could heat up more than the *p* legs, so we could distinguish them by their temperature difference due to the color scale presented in the 2D graphs.

**Figure 9:**
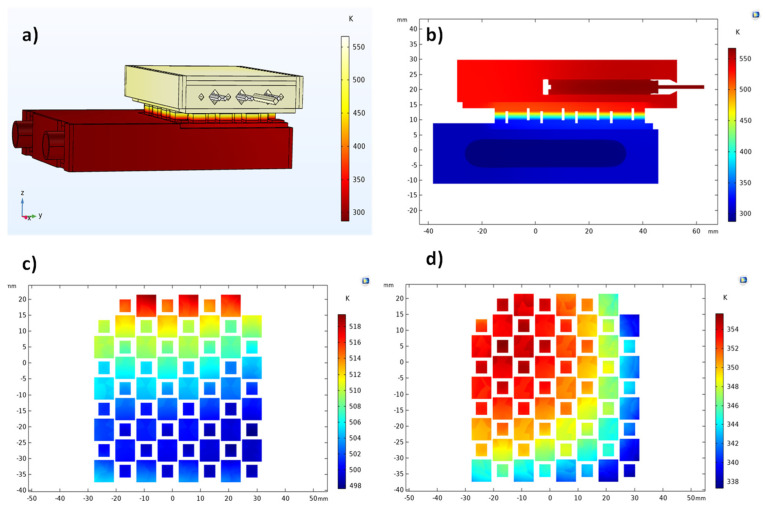
(**a**) Temperature distribution across the testing system when using silicide-tetrahedrite TEG with optimized geometry; (**b**) temperature distribution from a vertical plane cut taken from the middle of the plate; (**c**) temperature distribution on the top of the TEG legs (hot zone); (**d**) temperature distribution on the bottom of the TEG legs (cold zone).

The simulations of the silicide-tetrahedrite device with optimized geometry are shown in Figure 9. In this last case, the temperature between the TE legs, shown in Figure 9c, and 9d, was much more inhomogeneous than in previous cases, but the temperature variation was within the same order of magnitude (+/−20 K). Due to the geometry optimization, the difference in temperature observed between the *n* and *p* elements was much less evident than for the case where conventional geometry was simulated (Figure 8c). This effect verified the effectiveness of the system optimization, which was more thermally efficient, as shown in the simulations using the IFDM method (Figure 6).

**Figure 10:**
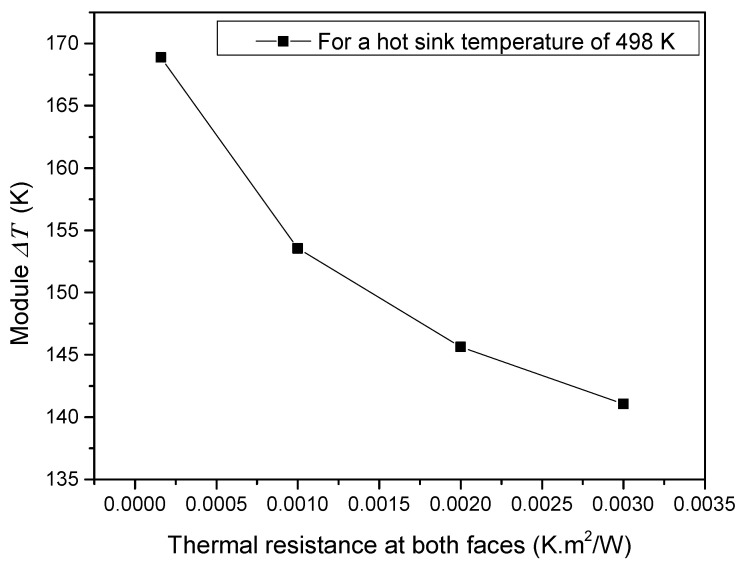
Influence of TEG surface thermal resistance on module Δ*T*. Simulations were performed using the GM200 device.

An important feature that can affect the performance of TE devices is the thermal contact resistance at the TEM faces. Despite often overlooked or over-rated, high thermal resistances can arise at the TEM faces if they are not properly installed. Therefore, to study the influence of TEM installation, simulations were performed using the M1 and M2 CAD models. In these simulations, the heat source temperature was fixed, and the thermal resistance at both TEG faces was changed (Figure 10). The objective was to mimic different application conditions of the thermal paste, which generated different thermal resistances and demonstrated how the system behaved as the contact thermal resistances increased. According to the results, the module Δ*T* decreased as the thermal resistance at the TEM faces increased. As a consequence, the power extracted was much lower than expected because the device voltage significantly dropped. These results indicated that it is critical to choose thermal pastes with good thermal conductivity and to properly install the TE devices; otherwise, the power extracted will not correspond to the expectations (or the device’s technical datasheet).

**Figure 11:**
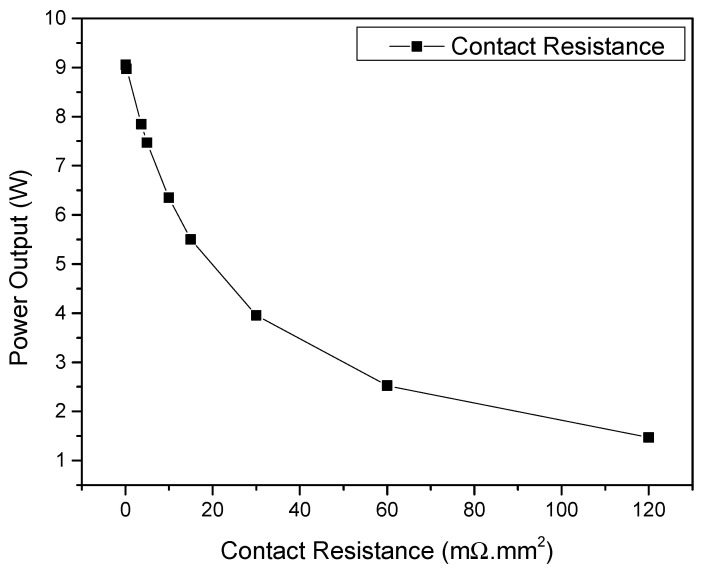
Contact resistance influence on the power produced by a silicide-tetrahedrite TEG (optimized geometry) for fixed hot- and cold-side temperatures of 568 and 321 K, respectively.

A similar effect was expected regarding the module’s electrical contact resistance. High electrical contact resistances at the leg junctions can limit the power output of a device and significantly decrease its conversion efficiency. To understand how different electrical contact resistances affected the performance of the silicide-tetrahedrite devices, simulations were performed using the 3D CAD model M4 while fixing the hot- and cold-side temperatures of the TEG faces and simultaneously varying the electrical contact resistances at the *n*- and *p*-leg junctions. The simulation results presented in Figure 11 clearly show that the lower the electric contact resistance, the higher the power produced by the device for a fixed temperature difference. If the electrical contact resistances were high enough, the power output of the device decreased more than ~85% of its maximum capability without changing the thermoelectric properties of the legs. These results highlight the importance of producing devices with low electric contact resistances and demonstrate that, independent of the material’s *zT*, having good electrical contacts is extremely important to produce commercially competitive devices. Currently, the reported state of the art electrical contact resistance values in tetrahedrite legs range from ~30 to 60 mΩ.mm^2^, while those of magnesium silicides can vary between ~1 and 20 mΩ.mm^2^, both depending on the TE materials’ chemical composition and the contact preparation techniques, methods, and joining materials used [32,41,42,43]. It is expected that with the constant investigation of new joining techniques and materials, the contact resistance values will decrease further in the future and eventually reach the values reported for commercial Bi_2_Te_3_ devices (~0.07–3.7 mΩ.mm^2^) [44,45,46].

## 4. Conclusions

In this work, two different computer methods were applied for the first time to evaluate silicide-tetrahedrite modules. The short computational time needed to simulate the performance of TEGs under specific working conditions using the IFDM with the 3D and 2D analyses provided by the FEM resulted in a deeper understanding of a TE system working with different TE devices. It was observed through the simulations that the output of the novel silicide-tetrahedrite TEGs not only matched but even surpassed that provided by conventional-material TEGs if geometrically optimized, used at higher ΔTs (not reachable by bismuth-telluride modules), and if good electrical contacts can be produced. Specifically, the optimized geometry TEG was able to generate 8.7 W for a 275 K temperature difference, slightly higher than the 8.5 W achieved by the bismuth-telluride module at its maximum allowable temperature. At the same time, the geometry optimization of such novel devices can be critical or even required to boost their usability without the need to increase their TE properties, especially if the devices are based on two completely different materials. Moreover, how the heat source and heat sinks are configured seems to be important for the TEGs’ operation and to provide a homogenous temperature distribution along the TEMs’ cross-section to explore their full potential, independent of working with conventional or novel TE materials.

Concerning the effects of the electrical and thermal contact resistances, they tend to be fairly overlooked or even disregarded in the literature. However, minimizing these two parameters can be extremely significant in achieving good performance when generating energy from waste heat. Because the thermal resistances can reduce the module’s ΔT and therefore their final power output, module installation needs to be suitable to ensure good heat transfer. Due to the aforementioned reasons, it is important to choose the most suitable thermal paste (the more conductive, the better) and to disperse it very well across the TEM surface to minimize the thermal resistance at both faces. As with thermal resistance, the electrical contact resistance can also be critical. While in commercial devices, the contact fabrication has been well-explored and is well-known, more studies are required on new TE materials. As shown by the FEM simulations, the electrical contact resistance in silicide-tetrahedrite legs can significantly reduce the power output for the same temperature gradients without changing the TE properties of the TEM legs. This means that new devices based in these emerging materials (or others) may only be competitive against the commercial ones if good electrical contacts can be manufactured.

## Data Availability

Not applicable.

## Supplementary Materials

The following supporting information can be downloaded at: https://www.mdpi.com/article/10.3390/mi13111915/s1, Figure S1: CAD geometry for models M3 and M4: (a) silicide-tetrahedrite TEM interior, (b) M3 model with the TEM inserted into the testing system, (c) TEM general overview (M4 model), and (d) meshed silicide-tetrahedrite device; Table S1: Dimensions and specifications used for the simulations of the tetrahedrite and magnesium silicide TEG with an optimized geometry (M4 model).
