# Supplementary material for: Computer Simulations of Silicide-Tetrahedrite Thermoelectric Generators
Source: Micromachines (Basel). 2022 Nov 5;13(11):1915. doi: 10.3390/mi13111915 (PMC9698283; doi:10.3390/mi13111915)
Supplement: Supplementary file 1 [file micromachines-13-01915-s001.zip › micromachines-2005119-supplementary.pdf]

micromachines

Supplementary Material

Computer Simulations of Silicide-tetrahedrite Thermoelectric Generators

Rodrigo Coelho [1], Álvaro Casi [2], Miguel Araiz [2], David Astrain [2], Elsa Branco Lopes [1], F. P. Brito [3] and António P. Gonçalves [1,*]

[1] C2TN, DECN, Instituto Superior Técnico, Universidade de Lisboa, Campus Tecnológico e Nuclear, 2695-066 Bobadela, Portugal
[2] Department of Engineering, Institute of Smart Cities, Public University of Navarre, Campus de Arrosadia s/n E-31006, Pamplona, Spain
[3] MEtRICs, DEM, Universidade do Minho, Guimarães, Portugal
* Correspondence: apg@ctn.tecnico.ulisboa.pt; Tel.: +351-219946182

For the simulations with the new TE materials two additional 3D CAD models were designed. They were called M3 and M4 and their geometry can be observed on the figure S1. The used specifications and dimensions for the silicide-tetrahedrite TEM with the optimized geometry are resumed in the table S1.

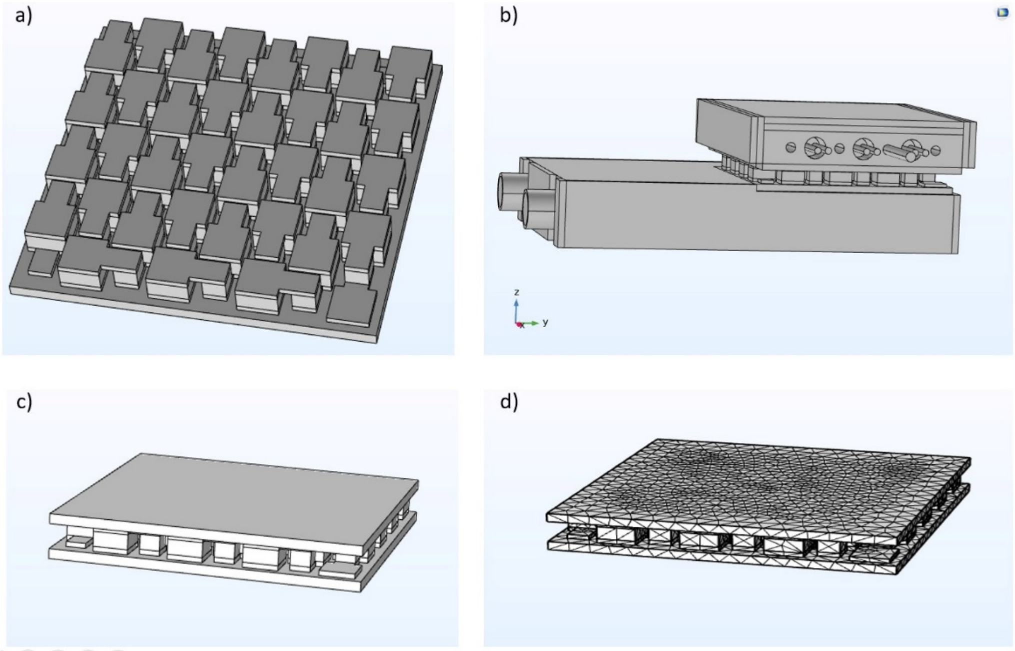

Figure S1. CAD geometry for the M3 and M4 models, a) Silicide-tetrahedrite TEM interior, b) M3 model with the TEM inserted into the testing system, c) TEM general overview (M4 model), and d) meshed silicide-tetrahedrite device.

Table S1. Dimensions and specifications used for the simulations of the tetrahedrite and magnesium silicide TEG with an optimized geometry (M4 model).

**Number of couples**	**35**
**Module height**	9 mm
**Alumina plates area**	62x62 mm
**Alumina Plates thickness**	2 mm
**Space between elements (*n* and *p*)**	1 mm on the *x* axis direction<br>2 mm on *y* axis direction
**TE elements size**	7x7 mm for type *p*<br>4x4 mm for type *n*
**TE elements height**	3 mm
**Copper electrodes thickness**	1 mm
**Copper electrodes surface area**	69 mm^2
**Materials used**	Tetrahedrite legs ($Cu_{11}Mn_1Sb_4S_{13}$) – type *p*<br>Magnesium silicide legs ($Mg_2(Si,Sn)$) – type *n*<br>Copper electrodes and alumina plates.
